# Supplementary material for: PERSIST platform provides programmable RNA regulation using CRISPR endoRNases
Source: Nat Commun. 2022 May 11;13:2582. doi: 10.1038/s41467-022-30172-3 (PMC9095627; doi:10.1038/s41467-022-30172-3)
Supplement: Supplementary file 1 — Supplementary Information [file 41467_2022_30172_MOESM1_ESM.pdf]

Supplemental Information

PERSIST: A programmable RNA regulation platform using CRISPR endoRNases

DiAndreth et al.

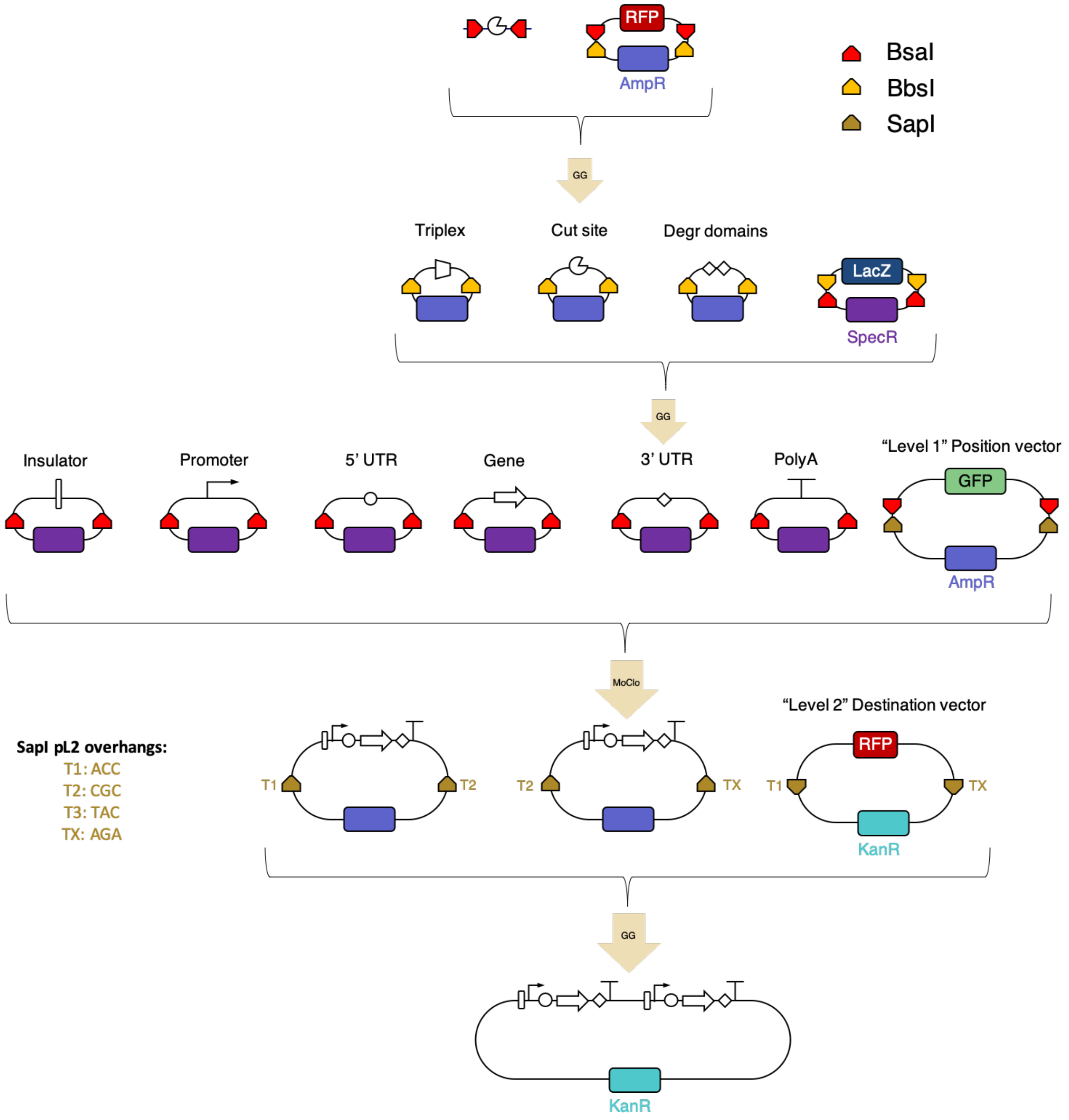

Supplementary Figure 1. Hierarchical golden gate cloning method. For the PERSIST-ON switch, sub-pL0s for each of the components were created using BsaI golden gate reactions into sub-pL0 backbones containing an ampicillin resistance gene. sub-pL0s were made for each of the three ON-switch components: triplex, cut site (endoRNase recognition site, miRNA target site, or ribozyme) and degradation domains. pL0[3'-UTR] position vectors were made through BbsI golden gate reactions with sub-pL0 vectors and pL0[3'-UTR] backbone vector containing a gene for spectinomycin resistance. pL1 expression vectors were made through BsaI golden gate reactions (MoClo [1]) with all six pL0 vectors and pL1 position vector backbone vector containing a gene for ampicillin resistance. Finally, multi-transcription unit pL2s are created through SapI golden gate reactions with pL1 vectors and pL2 backbone vector containing a gene for kanamycin resistance. Previously, this last pL2 step was often performed through Gibson assembly, but here we develop a SapI cloning scheme with the depicted overhangs which improves efficiency and streamlines the assembly process.

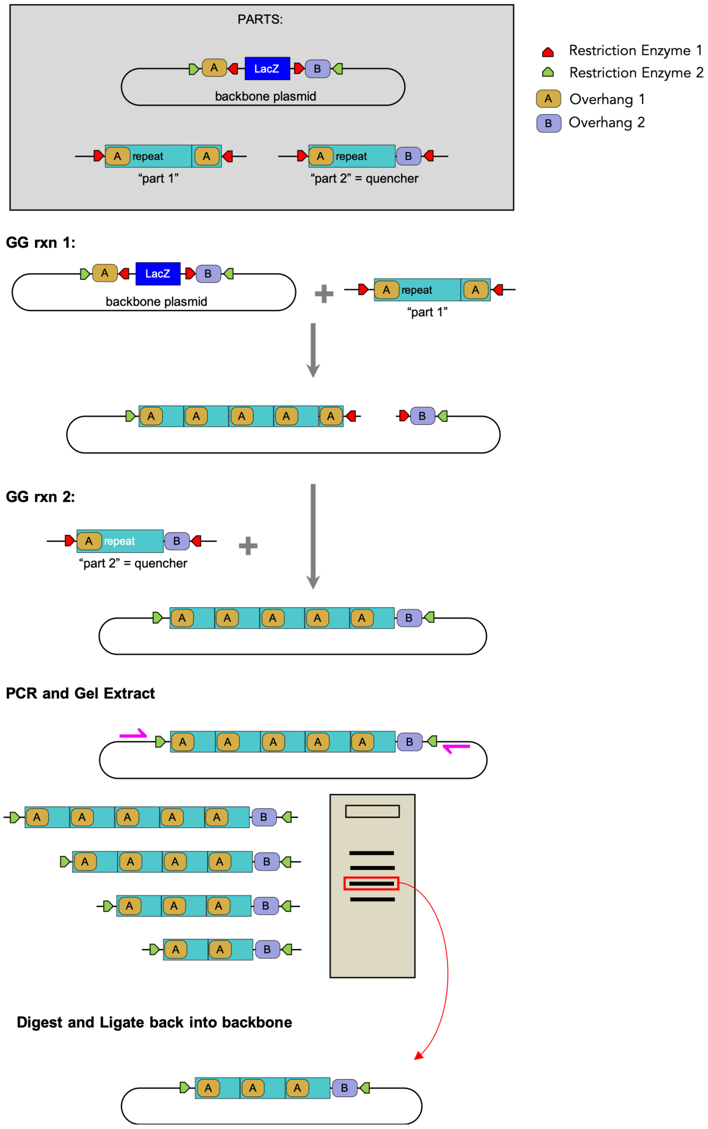

Supplementary Figure 2. "NxGG": In-house strategy for developing constructs with many sequence repeats. Briefly, repeat elements are designed to contain Type IIs restriction sites with the same overhangs on both sides ("part 1"). A Golden Gate reaction step with the backbone will allow part 1 to concatenate on the backbone. Adding the quencher, "part 2", binds to part 1 and the backbone to close the plasmid and complete the reaction. When directly transformed, the reaction has significant bias towards only one repeat so generally PCR was performed on the completed reaction mixture in order to extract the band of choice with selected number of repeats and re-digested and ligated back into the backbone. For the repeats of degradation sequences used here, both part 1 and part 2 consisted of 5 repeats of the wt1 sequence which was then concatenated.

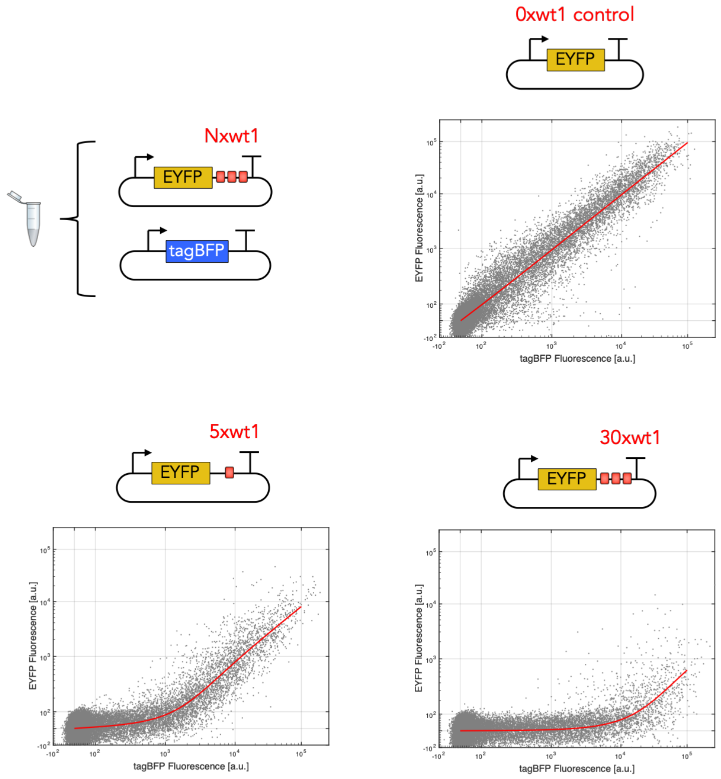

Supplementary Figure 3. **Data analysis for 1D co-transfection.** For experiments where only one transfection complex is made we measure reporter fluorescence (EYFP) as a function of transfection amount (tagBFP). For transcript degradation via wt1 degradation signals or by ribozyme cleavage, we assume that degradation machinery is not limiting and thus compute linear fits of the reporter and transfection marker fluorescence. This resulting fitted slope results in a holistic evaluation of transfection data which typically takes into account $>$ 10,000 cells and does not bias analysis based on transfection marker bin choice.

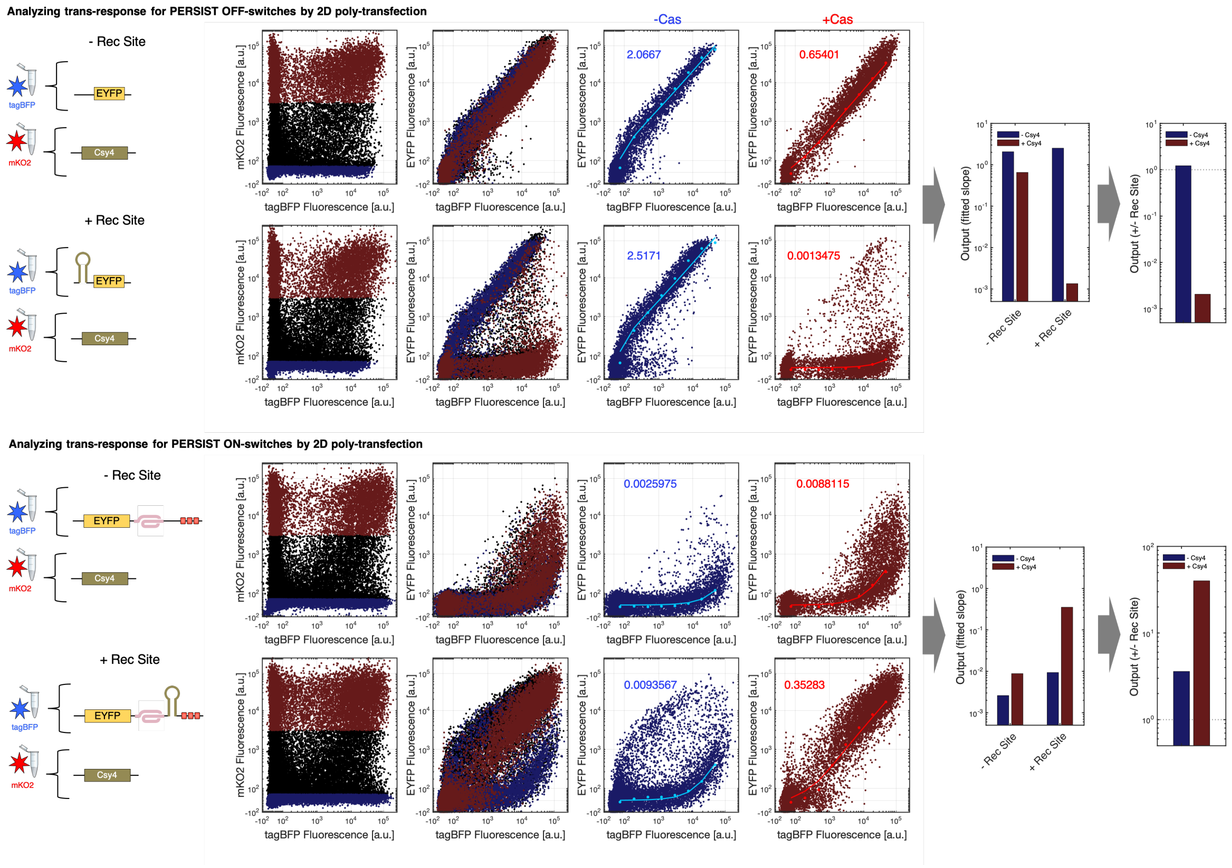

Supplementary Figure 4. Analysis of endoRNase PERSIST platform responses using 2D poly-transfection Here we demonstrate the evaluation of Csy4 in the PERSIST OFF- and PERSIST ON-switches. For either OFF- or ON-switch analysis of Csy4 response, switch constructs were compared to similar control constructs that did not contain the Csy4 recognition site (- Rec Site). Csy4 plasmid transfection efficiency was tracked by a plasmid encoding constitutive mKO2 while EYFP reporter plasmid transfection efficiencies were tracked by a plasmid encoding constitutive tagBFP. The first column shows the poly-transfection [2] scatter plots for each reporter with Csy4 which are similar across reporters and sample the 2D transfection space well. Data was then categorized as either having low (blue dots) or high (red dots) mKO2 fluorescence which is proportional to Csy4 plasmid transfection efficiency. A cutoff of below 50 for low mKO2 and above 3000 for high mKO2 was used for all 2D poly-transfection experiments. At high Csy4 values where the recognition site is present, we see an expected shift in population. Each of these populations were binned on tagBFP fluorescence and summary values were computed by fitting to an extreme value distribtion using the $evfit$ function in MATLAB. Linear fits of these summary values could then be evaluated as in the 1D calculations. Computed fits for each sample were then normalized to those for the control samples without the recognition site. Finally, fold changes could then be calculated for + Csy4/ - Csy4. This method therefore takes into account and normalizes for any non-specific effects observed by added endoRNase alone. This same method was also used to evaluate miRNA response as in Supplementary Figure 20.

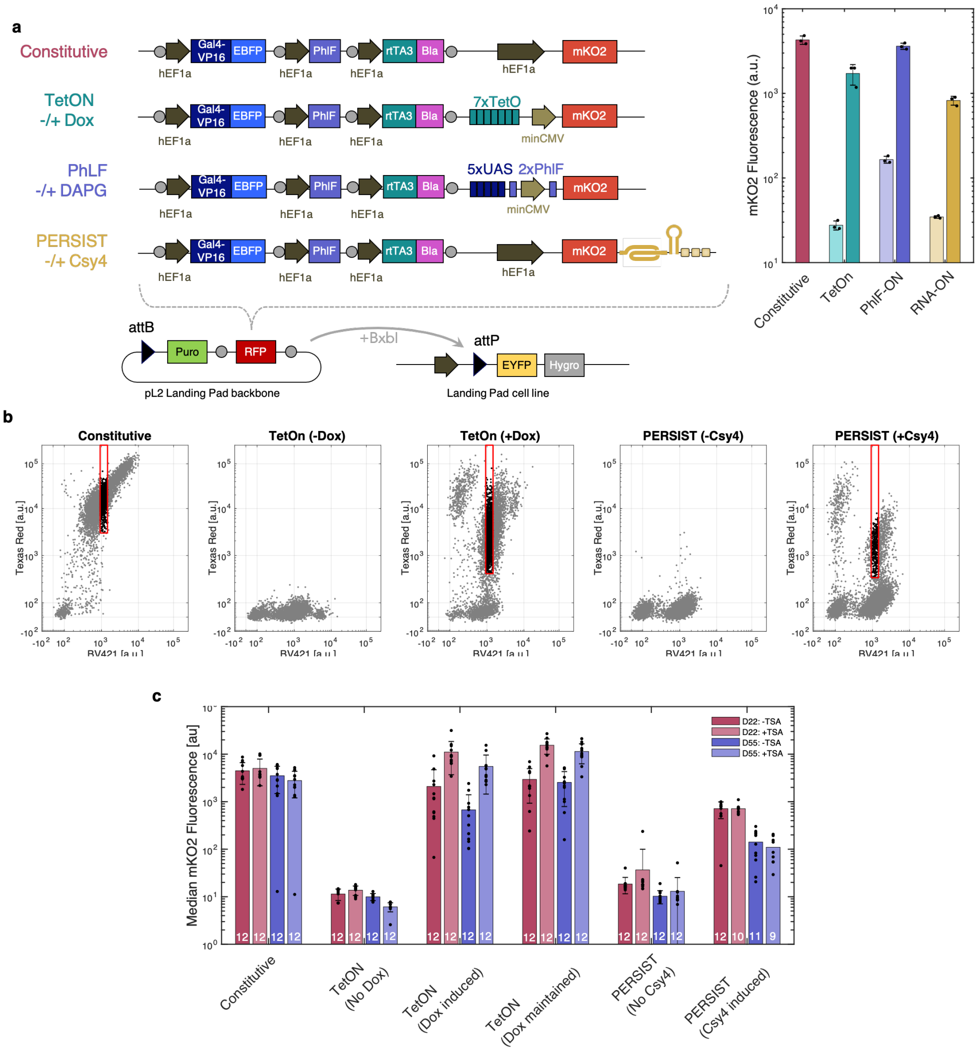

Supplementary Figure 5. Generating cell lines to evaluate silencing. a, Circuits for each switch were assembled into a pL2 vector containing necessary attB sites for landing pad integration. In order to compare switches, parts required for all circuits (Gal4-VP16, PhlF, and rtTA3) were cloned upstream of each switch in order to isolate any differences to the expression and silencing of the last transcription unit. Plasmids were integrated into Rosa26 LP CHO-K1 cell lines [3] using BxbI and under puromycin and blasticidin selection populations were seen that exhibited a shift from EYFP to EBFP implying the circuit was integrated successfully. Poly-clonal integrated lines were induced and evaluated for their response (right). The PhlF-On circuit did not behave as expected due to high background so was not continued for single-cell sorting. n=3 biologically independent samples where each sample represents the evaluation of $>$ 1,000 transfected cells. Data are presented as mean $\pm$ s.d. **b,** For single-cell sorting, the cell lines were induced with either Dox or Csy4 and compared to lines that were not induced to evaluate threshold for sorting. The following EBFP gate was used to sort all cell lines: Blue $>$ 925 and $<$ 1432. Importantly, cells were sorted only if they exhibited high response to induction to ensure a positive baseline for all samples. The following mKO2 threshold were used for each circuit: Constitutive $>$ 2922, TetOn $>$ 445, PERSIST $>$ 373. A 96-well plate of each cell line was collected, except for the Tet-On line where two plates were collected for which one was maintained in 4uM doxycycline and the other was not. **c,** 12 single-cell-sorted lines were selected randomly for further culturing and response evaluation. Cell lines were induced with either Dox or Csy4 and at the same time were evaluated in the presence or absence of 100nM TSA. Dots show median mKO2 fluorescence for cell lines that had at least 5 cells and bars represent mean $\pm$ s.d. of the number of biological replicates indicated in the bars. As shown both the Constitutive control circuit and PERSIST show little change in response to TSA addition, however the Tet-On system response to Dox is increased about 10-fold in the presence of TSA, implying that the cells are experiencing epigenetic silencing. Data are evaluated for the number of biologically independent samples indicated in the bars where each sample represents the evaluation of $>$ 5 cells. Data are presented as mean $\pm$ s.d.

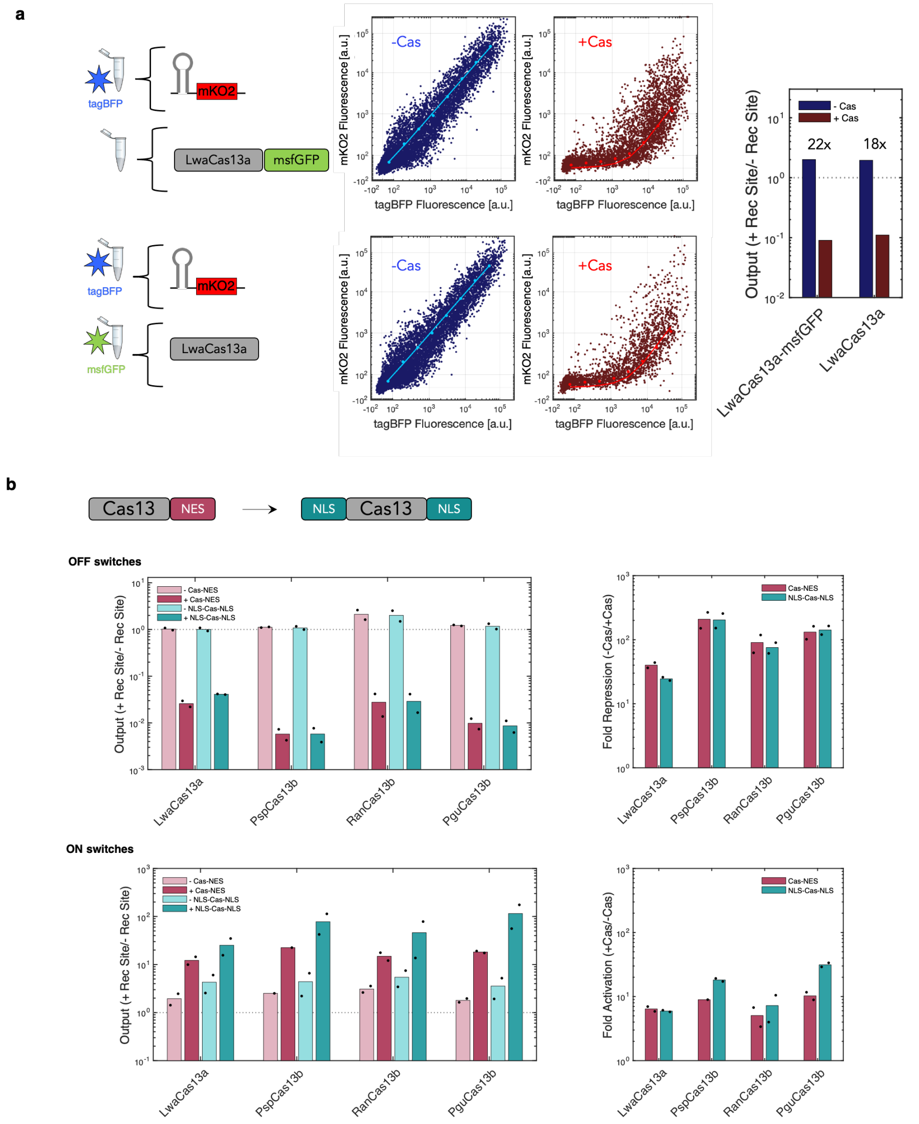

Supplementary Figure 6. Cas13 endoRNases engineering. a, Initial efforts carried out by Abudayyeh et al. [4] showed that msfGFP-tagged LwaCas13a behaved favorably for RNA-guided RNA cleaving. Here we test whether msfGFP is also necessary for the gRNA processing function and show that LwaCas13a without msfGFP tag performs similarly to the tagged protein. Shown are poly-transfection scatter plots where endoRNase was tracked with msfGFP and output fluorescence was evaluated by a mKO2 reporter and tracked by tagBFP. Fitting and quantification was otherwise carried out as in Supplementary Figure 4. $n \geq 1$ independent sample where each bar represents the evaluation of $>$ 1,000 transfected cells. **b,** Other than PfCas6 the Cas6 family of endonucleases have slightly improved performance compared to the Cas13 family. The Cas6 family of endonucleases are quite small, less than 30 kDa, which means that they can likely diffuse into the nucleus. We hypothesized that some of the success of these proteins in particular could be attributed to this ability since they are able to process transcripts before they have the chance to get translated in the cytoplasm. The Cas13 RNases are quite large, over 100 kDa, and all but Cas13d had been published (and tested here) with nuclear export signals. We evaluated whether nuclear localization could improve the performance of Cas13 family endoRNases by replacing the nuclear export sequences on these proteins with nuclear localization sequences on either end. Bar plots show normalized output values for each endoRNase. Nuclear localization showed a slight beneficial effect for several of the 3' ON switches but had little effect for OFF-switches. n=2 biologically independent samples where each sample represents the evaluation of $>$ 1,000 transfected cells. Data bars are presented as mean.

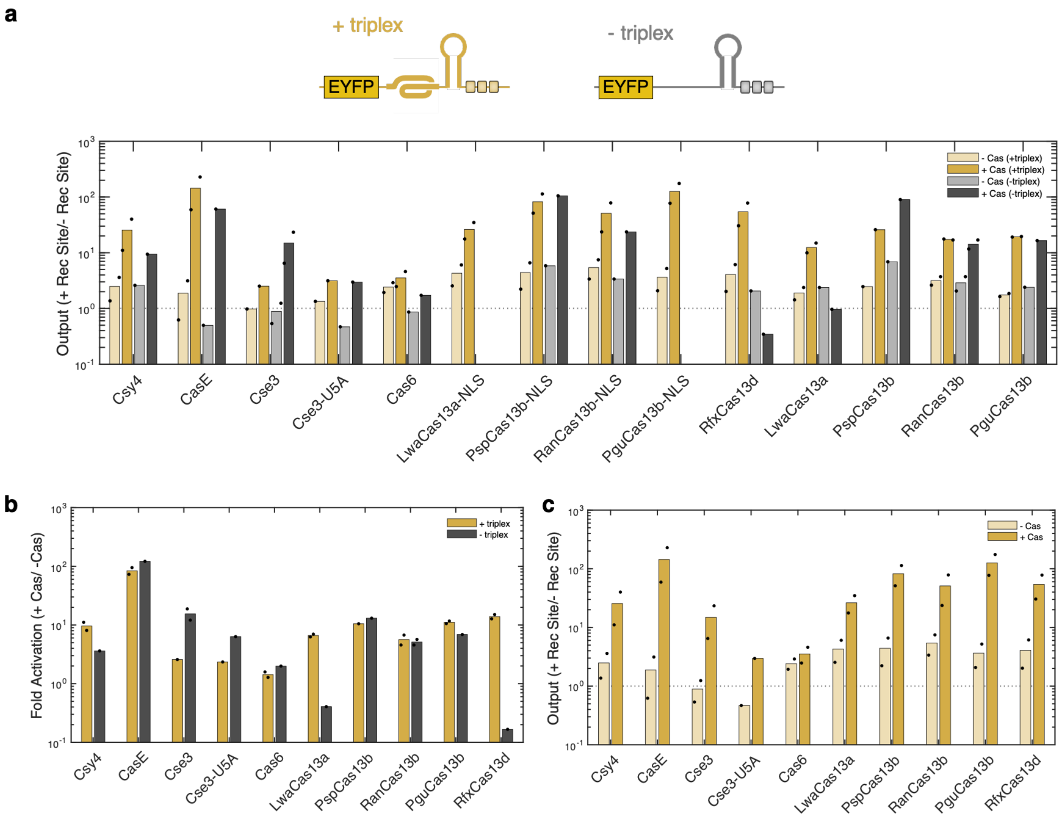

Supplementary Figure 7. Evaluating triplex requirement in PERSIST ON-switch. a, Reporters were made for each endoRNase that did or did not contain the MALAT1 triplex. Slopes were calculated as in Supplementary Figure 4. For some endoRNases (e.g. Cse3), removing the triplex resulted in either a decrease in fluorescence background or higher induction levels leading to a larger possible dynamic range. **b,** Fold activation values were calculated from (a) for the Cas6 endoRNases and the non-NLS engineered Cas13 variants showing larger dynamic range for Cse3 without the triplex. Note that for endoRNases that remained bound to the 3' cleaved product (i.e. LwaCas13a and RfxCas13d) the triplex was required for transcript stabilization post-cleavage. **a,b,** n=1 or n=2 biologically independent samples as indicated by the dots where each sample represents the evaluation of > 1,000 transfected cells. Data bars are presented as mean where more than one sample was evaluated. **c,** Optimal constructs were chosen for each endoRNase which consisted of reporters that contain triplexes for all endoRNases except Cse3 and Cas13 family endoRNases with NLS tags. Shown are normalized output values for the summary fold change values reported in Figure 1e. n=2 biologically independent samples where each sample represents the evaluation of > 1,000 transfected cells. Data bars are presented as mean.

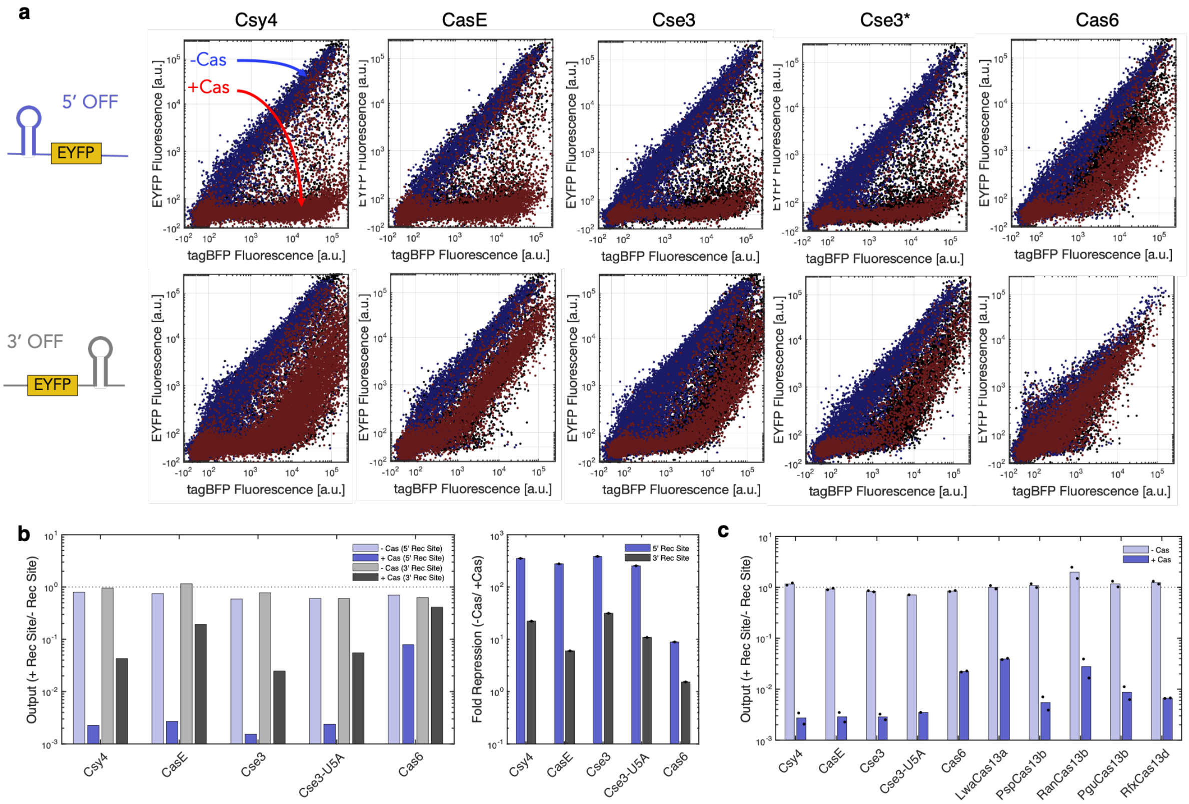

Supplementary Figure 8. **Evaluating endoRNase-responsiveness in PERSIST OFF switch. a,** For the Cas6 family of endoRNases cleavage sites were placed in either the 5'(top) or 3' (bottom) UTR to evaluate performance. Shown are scatter plots of cells with either high (red dots) or low (blue dots) Cas values as in Supplementary Figure 4. **b,** Summary fit values and fold change approximation were calculated for (a). For all cases, placing the endoRNase recognition site in the 5' UTR resulted in improved repression. This is likely because these endoRNases stay bound to the 5' cleaved product and would potentially protect the transcript from degradation in the 3' UTR as observed in the samples without triplex in Supplementary Figure 7. **a,b,** n=1 independent sample where each sample represents the evaluation of $>$ 1,000 transfected cells. **c,** Optimal constructs were chosen for each endoRNase which consisted of reporters that contain recognition sites in the 5' UTR for all endoRNases. Shown are normalized output values for the summary fold change values reported in Figure 1e. n=2 biologically independent samples where each sample represents the evaluation of $>$ 1,000 transfected cells. Data bars are presented as mean.

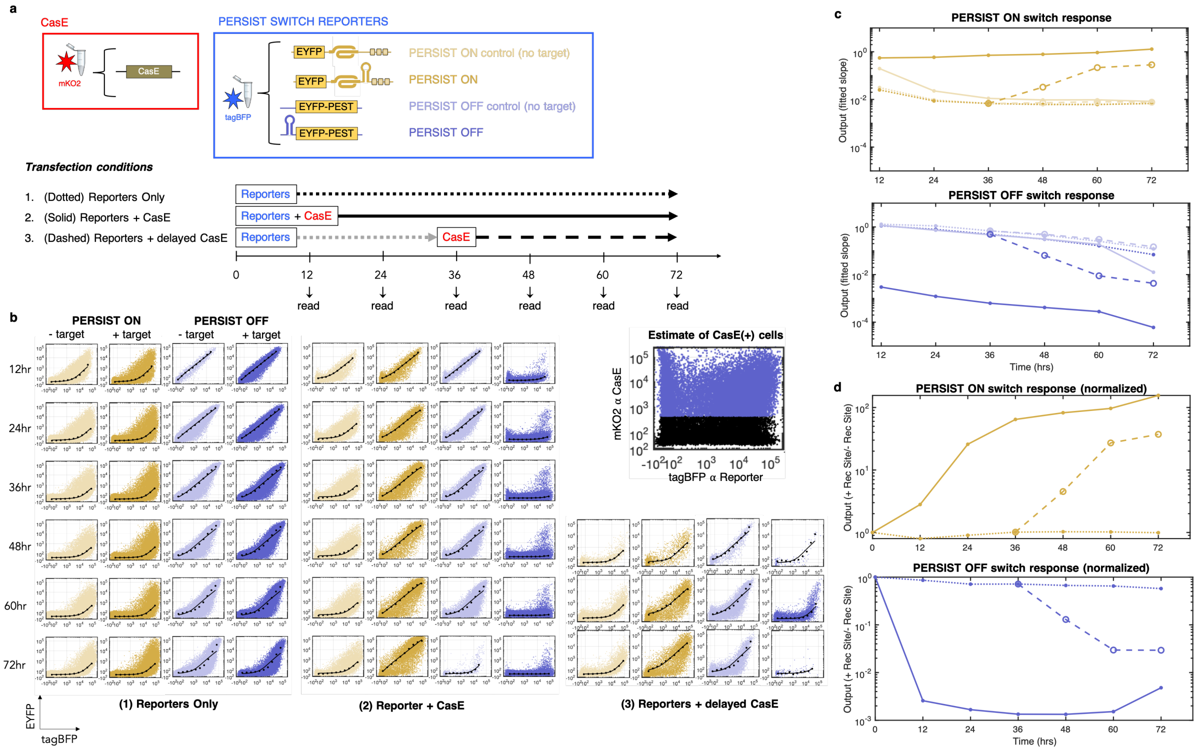

Supplementary Figure 9. PERSIST platform dynamics. a, Experimental workflow. PERSIST-ON and -OFF switch EYFP reporters (both CasE-responsive and non-target controls) were transiently transfected at t=0hr and tracked with tagBFP transfection marker. For each of these reporter conditions, CasE, tracked by mKO2 transfection marker, was transfected at t=0hr, t=36hr or not at all. Samples were performed in replicate so that wells could be evaluated by flow cytometry every 12 hours. Solid, dotted and dashed line notations are also used in plots shown in **c** and **d**. **b,** EYFP:tagBFP fitted slope values evaluated as in Supplementary Figure 4 for each reporter and CasE transfection condition. Here an mKO2 cut-off value of 500 was used to identify CasE-positive cells (shown in inset). **c,** Fitted slope values from (b) plotted over time for PERSIST ON- and OFF-switches (top and bottom respectively). **d,** Values from (c) for the PERSIST-ON and -OFF switch were normalized to their respective control reporters without target sites. For both switches, steady-state levels are reached within approximately 24 hours after CasE transfection which accounts for the delay in both CasE expression and PERSIST-switch response to CasE.

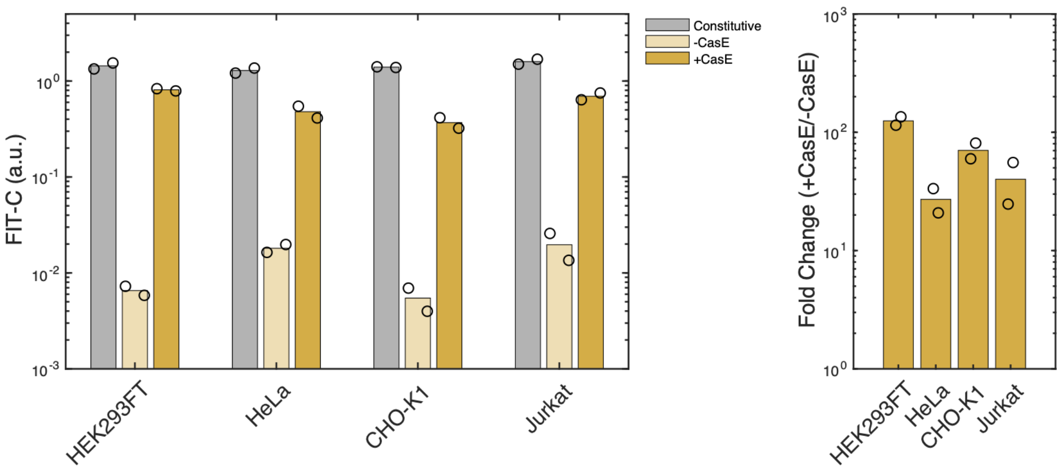

Supplementary Figure 10. PERSIST platform functions in a variety of cell types. The CasE-responsive PERSIST-ON switch EYFP reporter was transiently transfected and evaluated in four different cell types. All switches perform well where the switch is degraded effectively in the absence of CasE but approaches constitutive EYFP expression levels in the presence of CasE. n=2 biologically independent samples where each sample represents the evaluation of $>$ 1,000 transfected cells. Data bars are presented as mean.

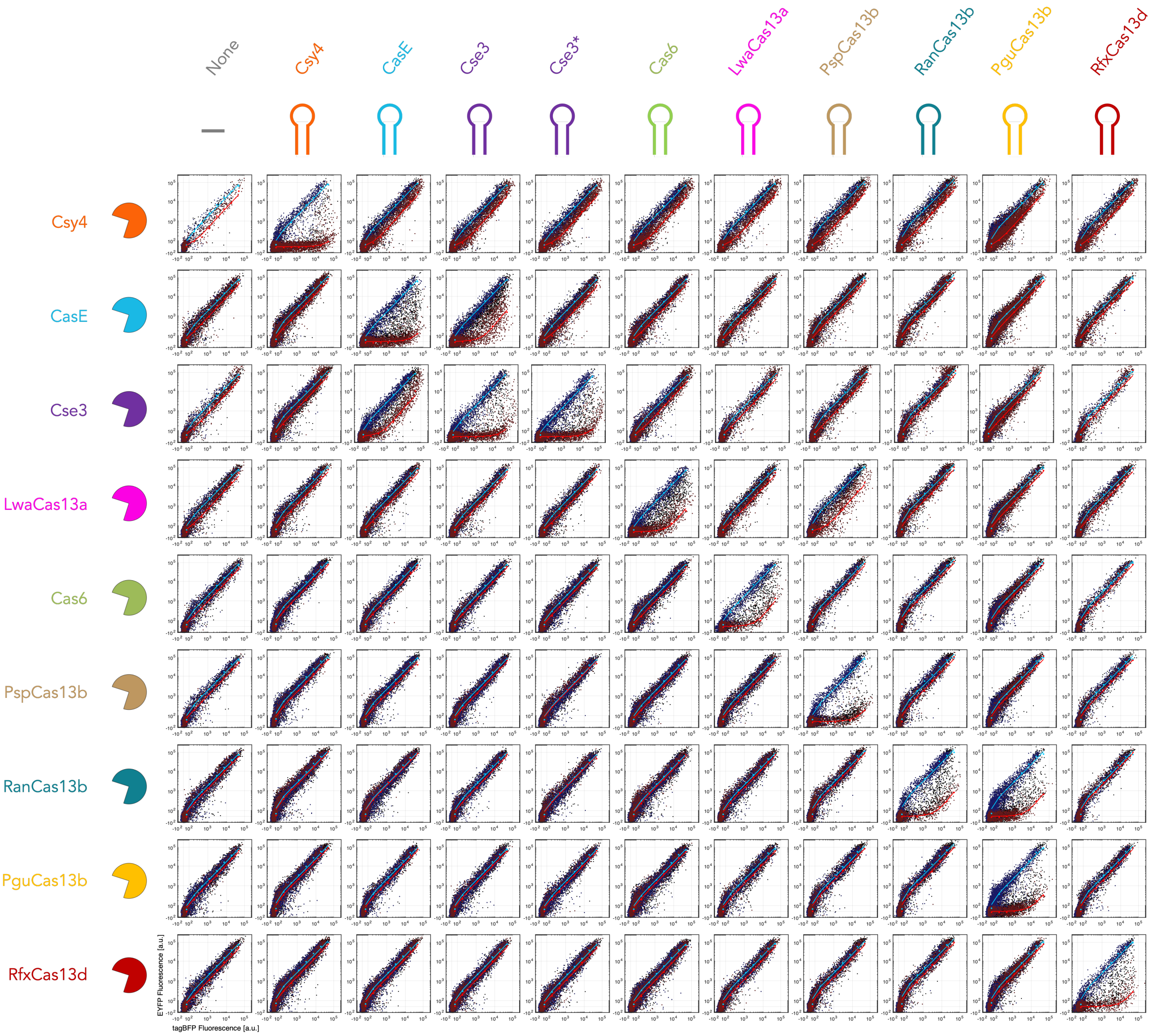

Supplementary Figure 11. Evaluating endoRNase orthogonality via poly-transfection PERSIST-OFF reporter and endoRNase pairs were evaluated via poly-transfection as in Supplementary Figure 4. Scatter plots show cells with either high (red dots) or low (blue dots) endoRNase values. n=1 biologically independent sample was evaluated for each combination where each sample represents the evaluation of > 1,000 transfected cells.

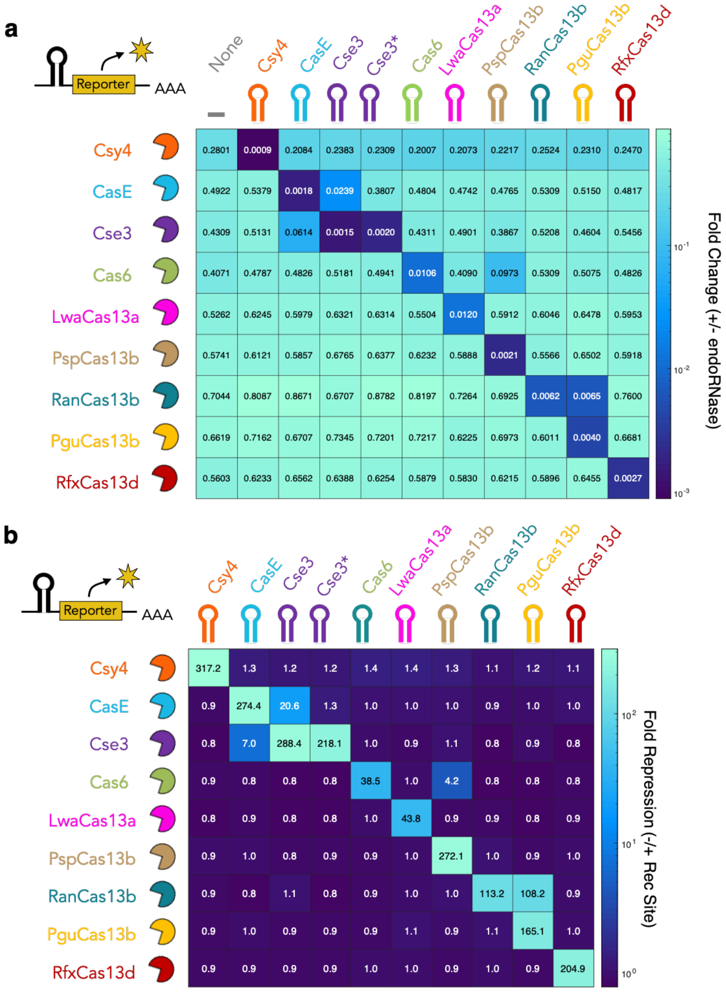

Supplementary Figure 12. Summary values of endoRNase orthogonality. a, Fold responses +/- endoRNase were computed from Supplementary Figure 11 as in Supplementary Figure 4 for each reporter. The computed values are shown in each square. Note off-target repression specifically for Csy4 across all reporters. **b,** Fold responses from (a) were normalized to the - rec site control ("None" column) to achieve the final fold repression values shown in Figure 2. The computed values are shown in each square. Here we see the overlap in cleavage between Cse3 and CasE which can be minimized using the mutated Cse3 hairpin (Cse3*).

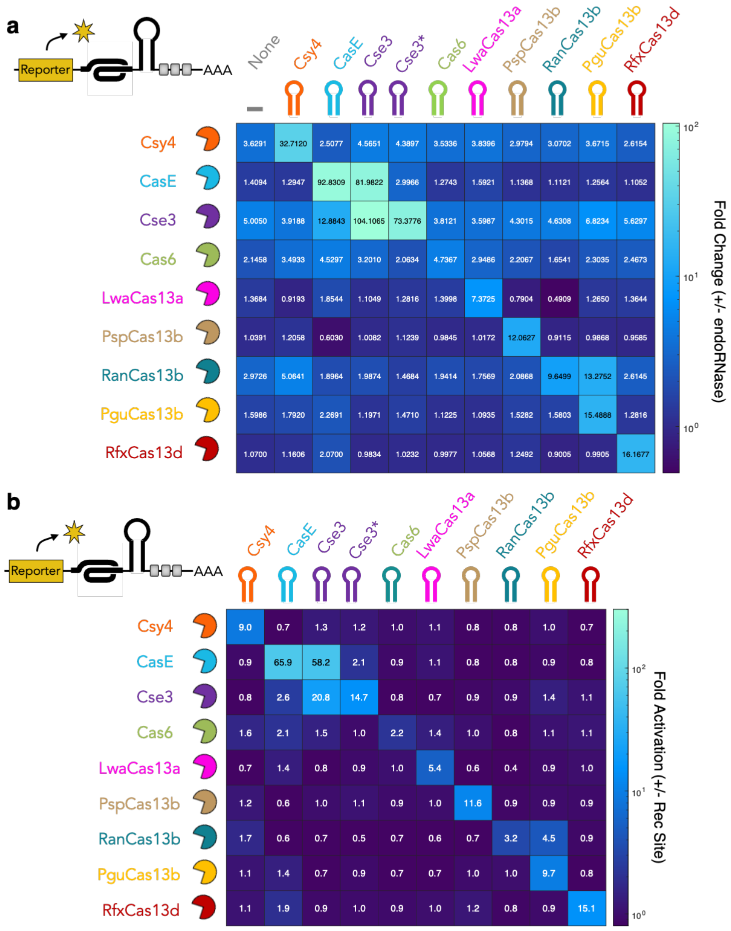

Supplementary Figure 13. Summary values of endoRNase orthogonality evaluated for PERSIST ON-switches. a, Fold responses +/- endoRNase were computed as in Supplementary Figure 12 for each reporter. The computed values are shown in each square. **b,** Fold responses from (a) were normalized to the - rec site control ("None" column) to achieve the final fold activation values. Orthogonality trends observed for PERSIST OFF-switches hold when evaluated by ON-switches as expected.

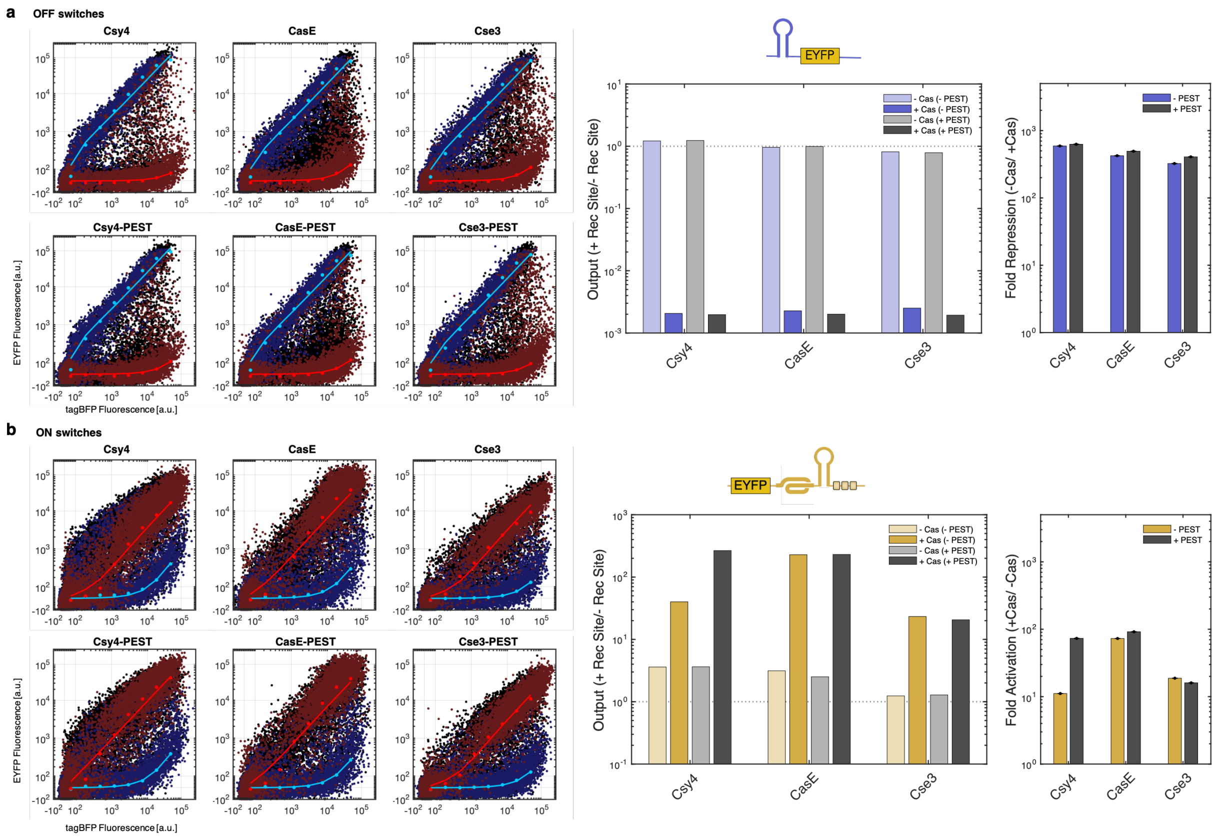

Supplementary Figure 14. Evaluating PEST-tagged endoRNases in PERSIST platform.
EndoRNase-responsive PERSIST OFF-switches **(a)** and ON-switches **(b)** were evaluated for Csy4, CasE and Cse with and without PEST tags. Scatter plots show cells with either high (red dots) or low (blue dots) endoRNase values and bar plots show summary output values calculated as in Supplementary Figure 4. PEST tags appear to have little effect on induction levels except for Csy4 where the PEST-tagged version appears to be beneficial for ON-switch rescue level, perhaps simply by reducing toxicity of Csy4. n=1 biologically independent sample was evaluated for each combination where each sample represents the evaluation of $>$ 1,000 transfected cells.

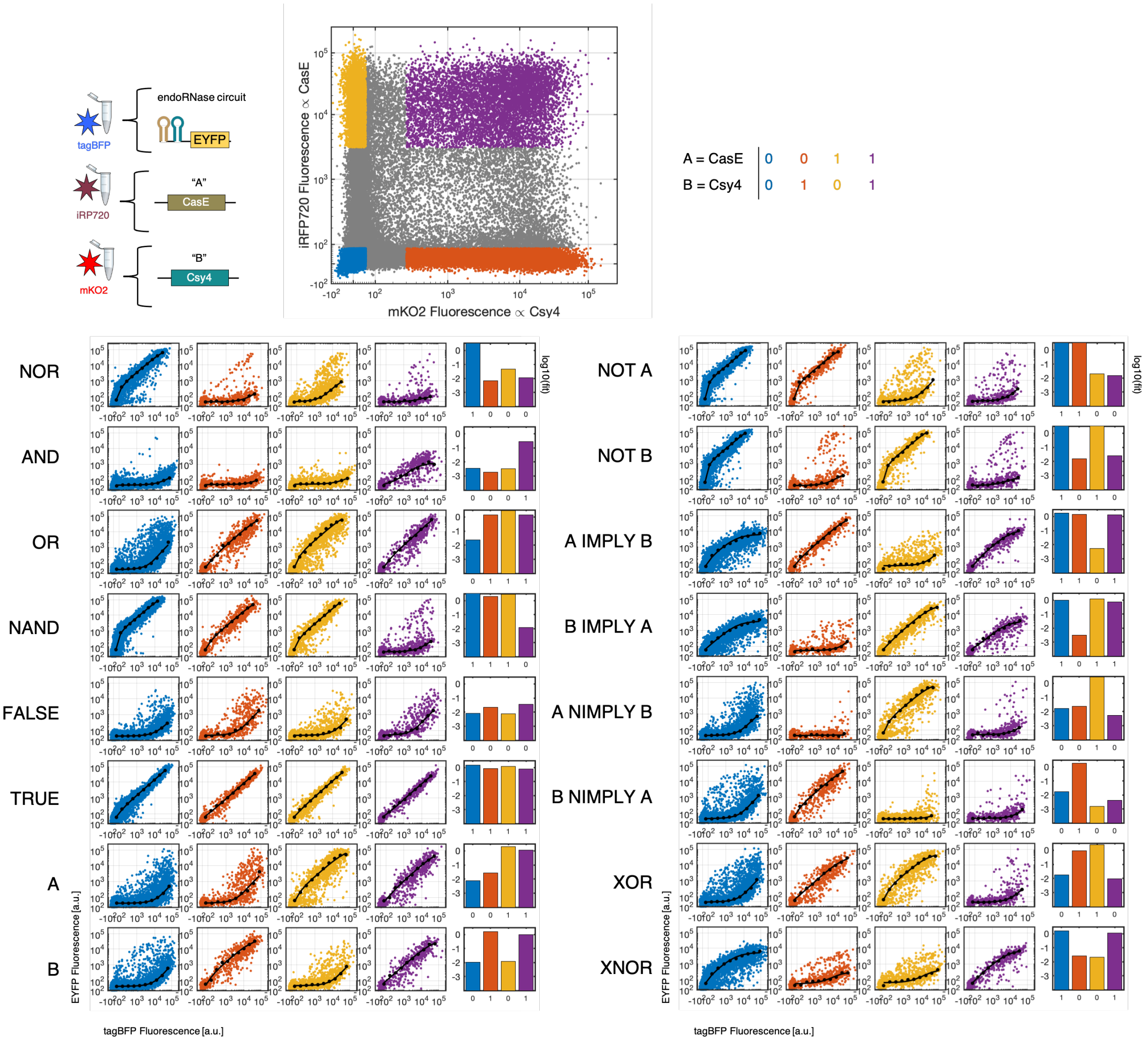

Supplementary Figure 15. Using poly-transfection for straightforward evaluation of logic functions Sample experimental approach and analysis for evaluating logic functions for a single representative sample. Plasmids encoding each logic function with appropriate EYFP reporter were transfected with a plasmid encoding tagBFP to track their transfection efficiency. Similarly, the amount of input endoRNases were tracked by fluorescent proteins: CasE by iRFP720 and Csy4 by mKO2. Poly-transfection resulted in a sampling of all combinations of input endoRNases which could be computationally isolated and analyzed. The following gates were used for each input combination: -CasE/-Csy4 (blue dots): iRFP720 $<$ 98th percentile of untransfected cells and mKO2 $<$ 98th percentile of untransfected cells, -CasE/+Csy4 (orange dots): iRFP720 $<$ 98th percentile of untransfected cells and mKO2 $>$ 300, +CasE/-Csy4 (yellow dots): iRFP720 $>$ 3000 and mKO2 $<$ 98th percentile of untransfected cells, and +CasE/+Csy4 (purple dots): iRFP720 $>$ 3000 and mKO2 $>$ 300. Scatter plots for each logic function show each isolated population which was fit in a similar fashion as in Supplementary Figure 4. However due to the composite nature of these devices made up of more than one transcript, we did not make the same linear assumption and instead fit these points to the full Michaelis-Menten saturation curve, $y = A \times X/(B + X)$. We then report the fitted value A/B in the bar plots. Devices exhibit expected outcomes indicated below each plot.

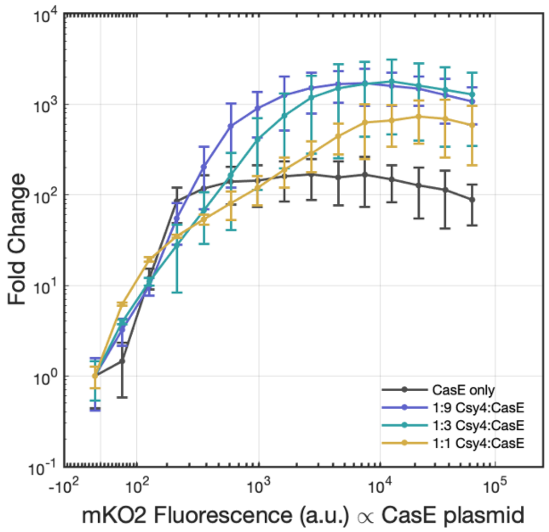

Supplementary Figure 16. cFFL fold change Fold change values calculated from Figure 4b. Fold change was calculated by normalizing to the EYFP value at the lowest mKO2 bin. Fold change evaluated at mKO2 = 10,000 is reported in the inset of Figure 4b. n=2 biologically independent samples were evaluated for each combination where each sample represents the evaluation of > 1,000 transfected cells. Data are presented as mean, error bars are ± s.d.

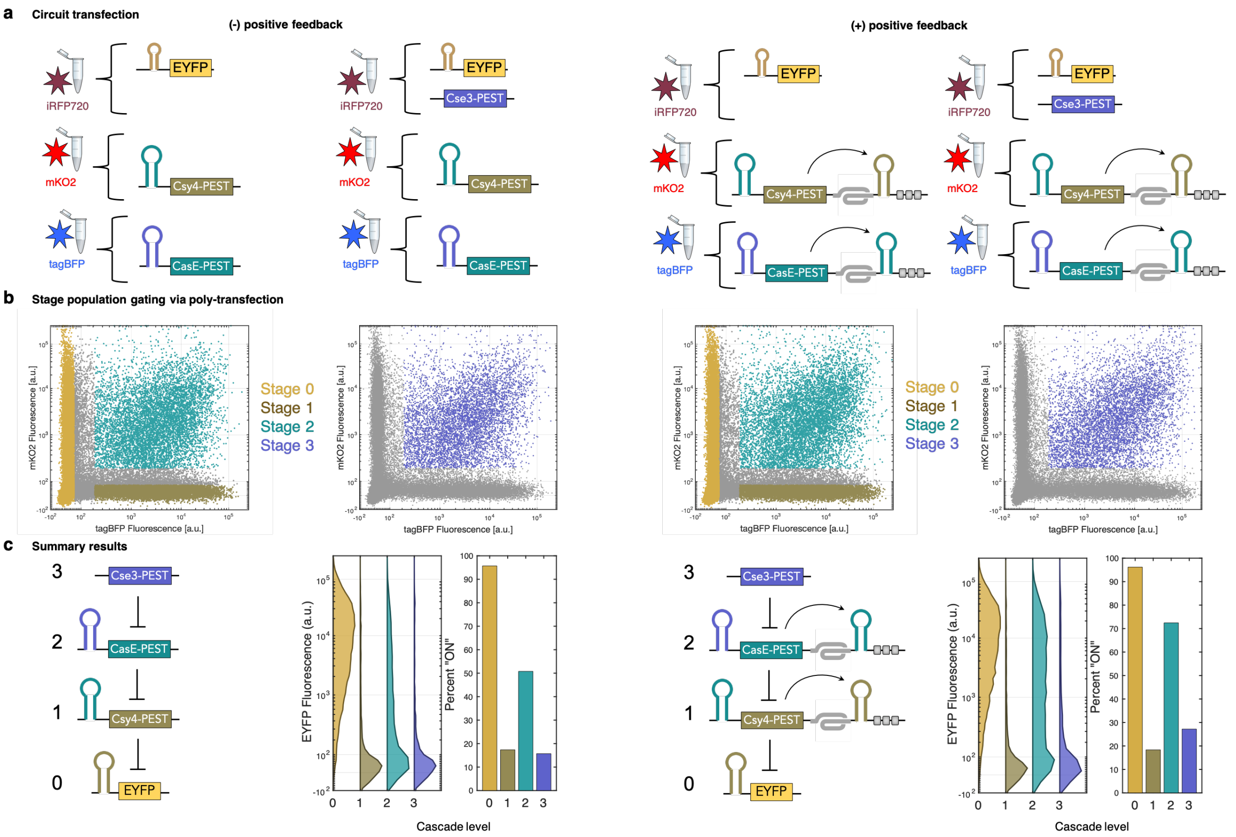

Supplementary Figure 17. **Evaluating 3-stage cascade** Sample experimental approach and analysis of a single representative sample for evaluating whether 3-stage repression cascade benefits from positive feedback. **a,** Poly-transfection was used to evaluate the stages of the cascade using different fluorescent proteins to track each stage's plasmid. The last stage (stage 3: Cse3) was combined with the reporter due to limits in the number of distinguishable colors and thus had to be included in a separate sample. **b,** Scatter plots for each poly-transfection show the populations that were isolated for each stage. The gating threshold for each population are as follows: stage 0: tagBFP $<$ 99 percentile of untransfected cells, stage 1: tagBFP $>$ 200 and mKO2 $<$ 99 percentile of untransfected cells, stage 2 and 3: tagBFP $>$ 200, mKO2 $>$ 200. For all evaluations only cells were evaluated that had iRFP $>$ 5000 to ensure highly-transfected reporter and Cse3 plasmid. **c,** EYFP fluorescence distribution is shown for each stage of the cascade and percent positive is quantified using a threshold of EYFP $>$ 200. Signal is restored from 50% to 70% in the second stage by using positive feedback with a trade-off in the third stage, which increases from 15% to 25%.

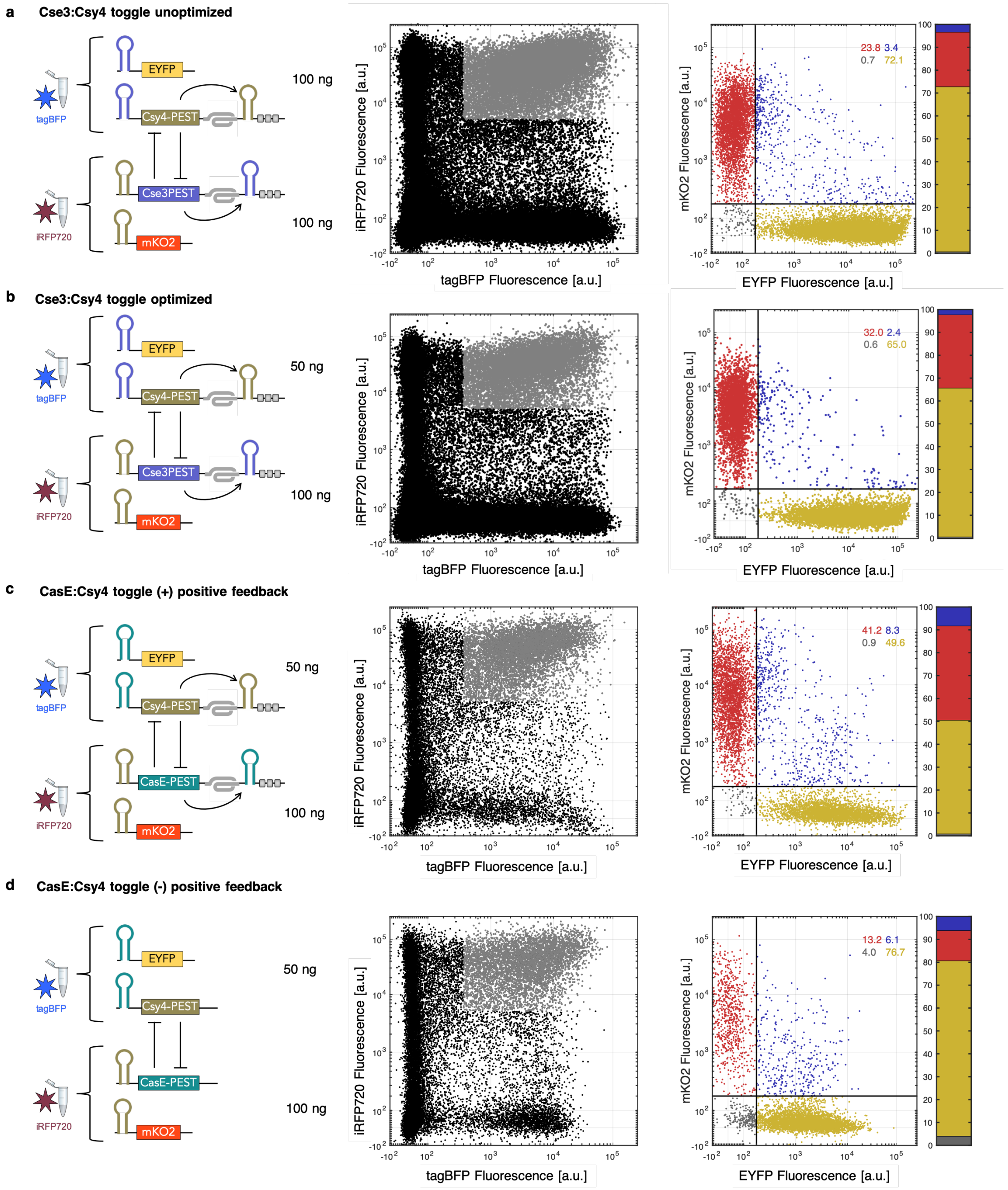

Supplementary Figure 18. Evaluation of various endoRNase-based toggle switches. For all switches, each endoRNase and corresponding reporter were tracked by a different fluorescent protein so that poly-transfection could be used to evaluate the bistable space. The following gates were used to evaluate cells that were transfected with both arms of the toggle: iRFP720 > 5000, tagBFP > 400. The following thresholds were used to designate cells as being in each state: mKO2 > 200, EYFP > 200 (black lines in rightmost plots). The bistable switch from Figure 5d was optimized from a version with equal amounts of each toggle arm transfected **(a)** to one with twice as much Cse3 arm **(b)**. Each bistable switch (rightmost plot) is shown with reference poly-transfection scatter plot showing the sampled region (left, gray dots). **c,** The same toggle switch topology can alternatively be built with CasE:Csy4. **d,** The same CasE:Csy4-based bistable switch without positive feedback has suboptimal bistability.

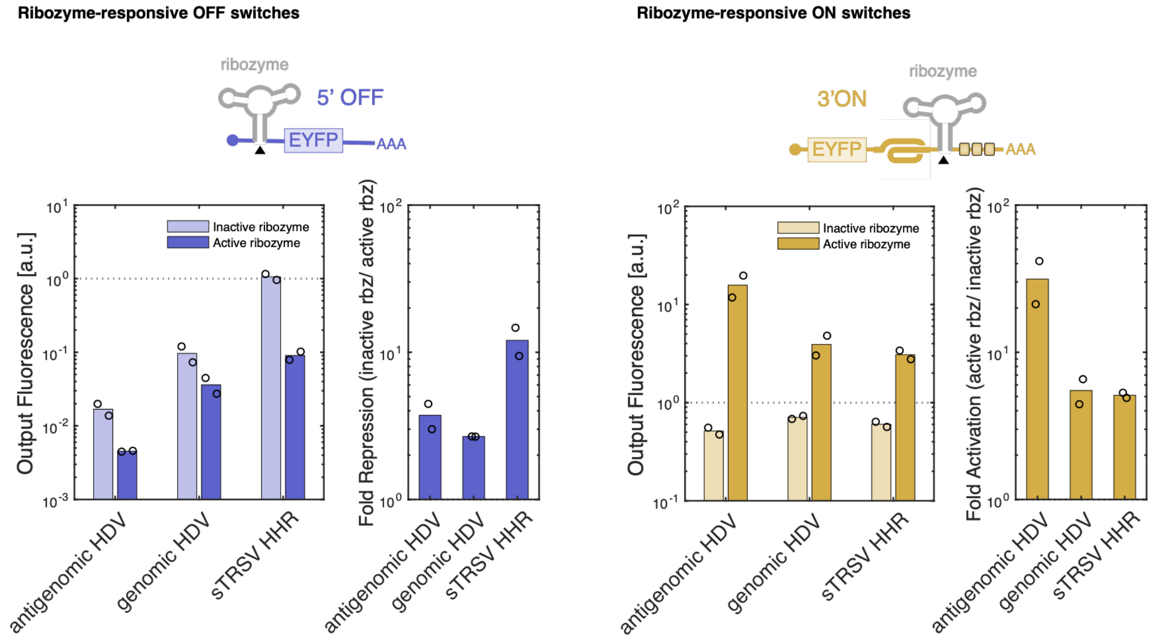

Ribozyme	Sequence
HDV Antigenomic	NGGGUCGGCAUGGCAUCUCCACCUCCUCGCGGUCCGACCUGGGCAUCCGAAGGAGGACGUACGUCCACUCGGAUGGCUAAGGGAG
HDV Antigenomic*	NGGGUCGGCAUGGCAUCUCCACCUCCUCGCGGUCCGACCUGGGCAUCCGAAGGAGGACGUACGUCCACUCGGAUGGUUAAGGGAG
HDV Genomic	NGGCCGGCAUGGUCCCAGCCUCCUCGCUGGCGCCGGCUGGGCAACAUGCUUCGGCAUGGCGAAUGGGAC
HDV Genomic*	NGGCCGGCAUGGUCCCAGCCUCCUCGCUGGCGCCGGCUGGGCAACAUGCUUCGGCAUGGUGAAUGGGAC
sTRSV	GCUGUCACCGGAUGUGCUUUCCGGUCUGAUGAGUCCGUGAGGACGAAACAGC
sTRSV*	GCUGUCACCGGAUGUGCUUUCCGGUCUGAUGAGUCCGUGAGGACGAGACAGC

Supplementary Figure 19. Evaluating ribozyme-responsiveness in PERSIST. Three different self-cleaving ribozymes (HDV antigenomic, HDV genomic, and sTRSV hammerhead) and each of their inactive versions were evaluated in either the 5' PERSIST OFF-switch (left) or 3' PERSIST ON-switch (right) positions. Shown are fitted fluorescent values calculated as in Supplementary Figure 4 normalized to analogous reporter constructs containing no ribozyme as well as normalized output values. For ribozymes in the 5' PERSIST OFF-switch position even some inactive ribozymes had large detrimental effects on expression where all showed additional repression when using an active ribozyme. For ribozymes in the 3' PERSIST ON-switch position, inactive ribozymes had little effect on the ability of the ON-switch motif to degrade transcripts and all active ribozymes increased reporter expression compared to their inactive versions. Table shows ribozyme sequences where red underline denotes the bases between which the ribozyme cleaves and blue underline denotes the inactivating mutation. n=2 biologically independent samples where each sample represents the evaluation of $>$ 1,000 transfected cells. Data bars are presented as mean.

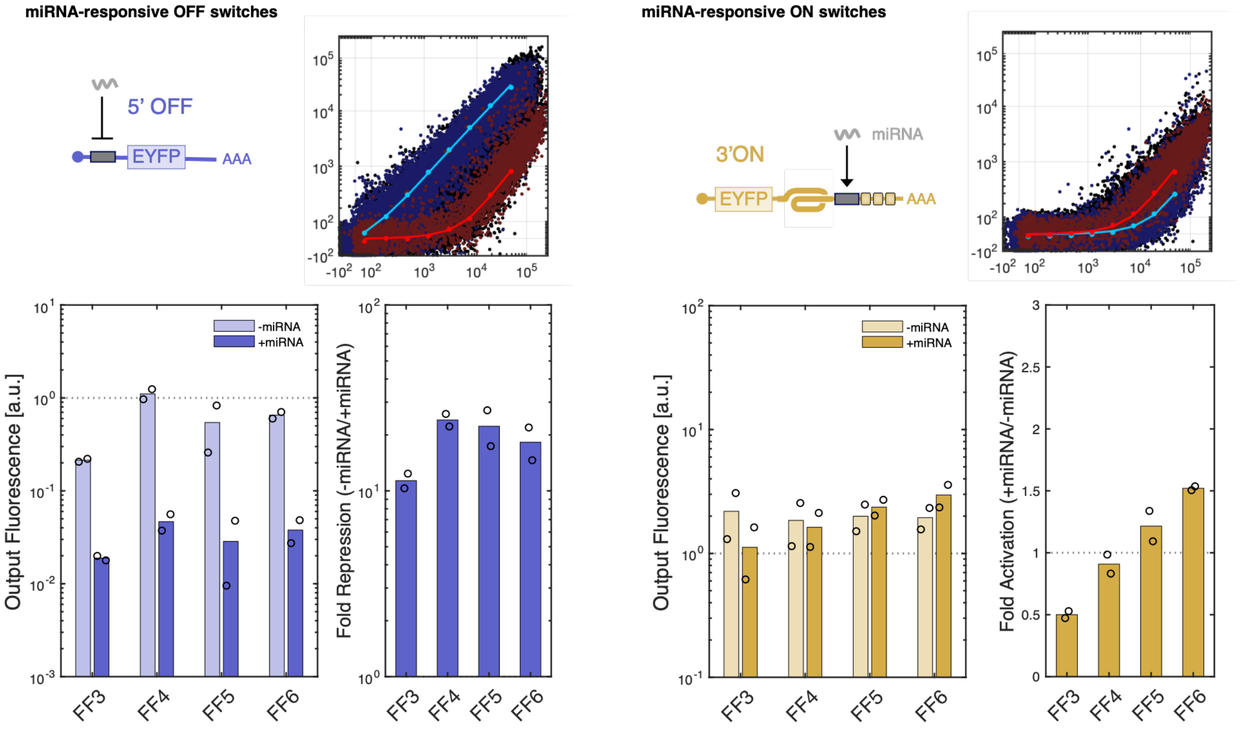

Supplementary Figure 20. **Evaluating miRNA-responsiveness in PERSIST.** Scatter plots show cells with either high (red dots) or low (blue dots) miRNA values as in Supplementary Figure 4. Bars below are normalized output values with single miRNA target sites placed in either the 5' PERSIST OFF-switch (left) or 3' PERSIST ON-switch (right) positions. Corresponding miRNAs were co-transfected and response was evaluated as in Supplementary Figure 4. All miRNAs resulted in repression in the PERSIST OFF switch as has been shown previously [5] and, promisingly, some miRNAs resulted in activation via the PERSIST ON switch, albeit modest in performance. n=2 biologically independent samples where each sample represents the evaluation of > 1,000 transfected cells. Data bars are presented as mean.

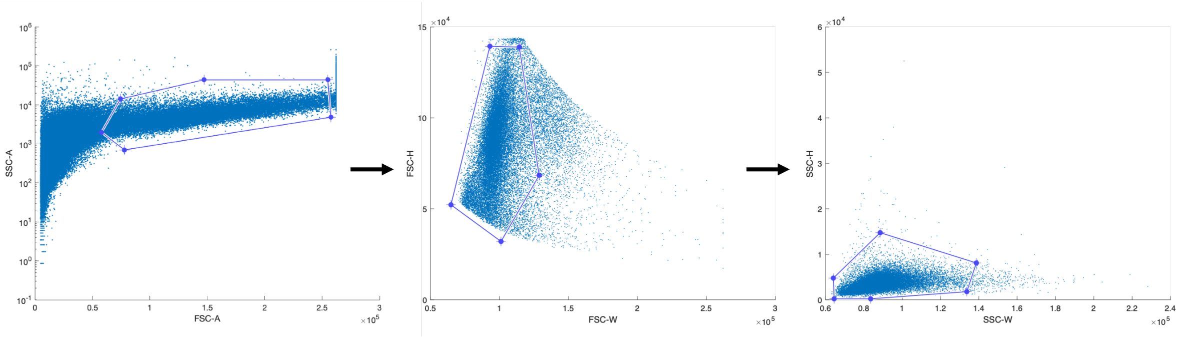

Supplementary Figure 21. Example gating strategy. Scatter plots show the gating strategy used to evaluate live cells for all transfection experiments.

REFERENCES

[1] Xavier Duportet, Liliana Wroblewska, Patrick Guye, Yinqing Li, Justin Eyquem, Julianne Rieders, Tharathorn Rimchala, Gregory Batt, and Ron Weiss. A platform for rapid prototyping of synthetic gene networks in mammalian cells. *Nucleic Acids Research*, 42(21):13440–13451, 2014.

[2] Jeremy J Gam, Breanna DiAndreth, Ross D Jones, Jin Huh, and Ron Weiss. A 'poly-transfection' method for rapid, one-pot characterization and optimization of genetic systems. *Nucleic acids research*, 47(18):e106, 10 2019.

[3] Leonid Gaidukov, Liliana Wroblewska, Brian Teague, Tom Nelson, Xin Zhang, Yan Liu, Kalpana Jagtap, Selamawit Mamo, Wen Allen Tseng, Alexis Lowe, Jishnu Das, Kalpanie Bandara, Swetha Baijuraj, Nevin M Summers, Timothy K Lu, Lin Zhang, and Ron Weiss. A multi-landing pad DNA integration platform for mammalian cell engineering. *Nucleic Acids Research*, 46(8):4072–4086, 2018.

[4] Omar O Abudayyeh, Jonathan S Gootenberg, Silvana Konermann, Julia Joung, Ian M Slaymaker, David B.T. Cox, Sergey Shmakov, Kira S Makarova, Ekaterina Semenova, Leonid Minakhin, Konstantin Severinov, Aviv Regev, Eric S Lander, Eugene V Koonin, and Feng Zhang. C2c2 is a single-component programmable RNA-guided RNA-targeting CRISPR effector. *Science*, 353(6299):aaf5573, 8 2016.

[5] Jeremy J. Gam, Jonathan Babb, and Ron Weiss. A mixed antagonistic/synergistic miRNA repression model enables accurate predictions of multi-input miRNA sensor activity. *Nature Communications*, 9(1):2430, 12 2018.
