# Supplementary material for: PERSIST platform provides programmable RNA regulation using CRISPR endoRNases
Source: Nat Commun. 2022 May 11;13:2582. doi: 10.1038/s41467-022-30172-3 (PMC9095627; doi:10.1038/s41467-022-30172-3)
Supplement: Supplementary file 2 — Reporting Summary [file 41467_2022_30172_MOESM2_ESM.pdf]

nature portfolio

Corresponding author(s): Ron Weiss

Last updated by author(s): April 5, 2022

Reporting Summary

Nature Portfolio wishes to improve the reproducibility of the work that we publish. This form provides structure for consistency and transparency in reporting. For further information on Nature Portfolio policies, see our Editorial Policies and the Editorial Policy Checklist.

Statistics

For all statistical analyses, confirm that the following items are present in the figure legend, table legend, main text, or Methods section.

n/a	Confirmed	
[ ]	[x]	The exact sample size (*n*) for each experimental group/condition, given as a discrete number and unit of measurement
[ ]	[x]	A statement on whether measurements were taken from distinct samples or whether the same sample was measured repeatedly
[x]	[ ]	The statistical test(s) used AND whether they are one- or two-sided<br>*Only common tests should be described solely by name; describe more complex techniques in the Methods section.*
[x]	[ ]	A description of all covariates tested
[ ]	[x]	A description of any assumptions or corrections, such as tests of normality and adjustment for multiple comparisons
[ ]	[x]	A full description of the statistical parameters including central tendency (e.g. means) or other basic estimates (e.g. regression coefficient) AND variation (e.g. standard deviation) or associated estimates of uncertainty (e.g. confidence intervals)
[x]	[ ]	For null hypothesis testing, the test statistic (e.g. *F*, *t*, *r*) with confidence intervals, effect sizes, degrees of freedom and *P* value noted<br>*Give P values as exact values whenever suitable.*
[x]	[ ]	For Bayesian analysis, information on the choice of priors and Markov chain Monte Carlo settings
[x]	[ ]	For hierarchical and complex designs, identification of the appropriate level for tests and full reporting of outcomes
[x]	[ ]	Estimates of effect sizes (e.g. Cohen's *d*, Pearson's *r*), indicating how they were calculated

Our web collection on statistics for biologists contains articles on many of the points above.

Software and code

Policy information about availability of computer code

Data collection	FACSDiva version 8.0.1 software was used to collect flow cytometry data.
Data analysis	Excel Version 16.40, Geneious Prime 2019.0.4, MATLAB 2019a. Custom MATLAB code was used for flow cytometry data analysis and plot generation and will be made available upon request.

For manuscripts utilizing custom algorithms or software that are central to the research but not yet described in published literature, software must be made available to editors and reviewers. We strongly encourage code deposition in a community repository (e.g. GitHub). See the Nature Portfolio guidelines for submitting code & software for further information.

Data

Policy information about availability of data

All manuscripts must include a data availability statement. This statement should provide the following information, where applicable:

- Accession codes, unique identifiers, or web links for publicly available datasets
- A description of any restrictions on data availability
- For clinical datasets or third party data, please ensure that the statement adheres to our policy

Representative plasmid sequences have been deposited to Genbank with the accession codes OM256462-OM256466.
All plasmids used in this study are described in this paper and sequence information for parts are provided. Additional sequence information are available upon reasonable request from the authors. Raw .fcs files are available upon reasonable request. Source data are provided with this paper.

Field-specific reporting

Please select the one below that is the best fit for your research. If you are not sure, read the appropriate sections before making your selection.

- [x] Life sciences
- [ ] Behavioural & social sciences
- [ ] Ecological, evolutionary & environmental sciences

For a reference copy of the document with all sections, see nature.com/documents/nr-reporting-summary-flat.pdf

Life sciences study design

All studies must disclose on these points even when the disclosure is negative.

Sample size	No sample size calculations were performed. Sample sizes were chosen to align with what is typical in the field.
Data exclusions	In Fig 1d, samples were excluded that contained fewer than 6 cells in order to ensure that summary values represented population responses. Of the 12 cell line clones for each construct type, only up to 2 clones needed to be excluded. The threshold value of 6 cells was used for a CV of 40%.
Replication	All attempts at replication were successful and at least two independent experiments were performed at least two days apart.
Randomization	Most experiments were performed on immortalized cell lines, which were uniformly allocated to individual sample wells for transfection. For single cell sorting, an identical number of single cells were collected for each construct/treatment type. For the TetOn construct, plates of cells were randomly assigned to the treatment (+DOX) group or non-treatment (-DOX) group. 12 clones were picked randomly from each construct group for further evaluation.
Blinding	Investigators were not blinded to allocation during experiments due to limitations in personnel required to separately carry-out sample preparation and data collection.

Reporting for specific materials, systems and methods

We require information from authors about some types of materials, experimental systems and methods used in many studies. Here, indicate whether each material, system or method listed is relevant to your study. If you are not sure if a list item applies to your research, read the appropriate section before selecting a response.

Materials & experimental systems

n/a	Involved in the study	
x		Antibodies
	x	Eukaryotic cell lines
x		Palaeontology and archaeology
x		Animals and other organisms
x		Human research participants
x		Clinical data
x		Dual use research of concern

Methods

n/a	Involved in the study	
x		ChIP-seq
	x	Flow cytometry
x		MRI-based neuroimaging

Eukaryotic cell lines

Policy information about cell lines

Cell line source(s)	HEK-293FT cells: Thermo Fisher, CHO-K1 cells: ATCC
Authentication	None of the cell lines were authenticated.
Mycoplasma contamination	All cell lines tested negative for mycoplasma.
Commonly misidentified lines (See ICLAC register)	None

Flow Cytometry

Plots

Confirm that:

- [x] The axis labels state the marker and fluorochrome used (e.g. CD4-FITC).
- [x] The axis scales are clearly visible. Include numbers along axes only for bottom left plot of group (a 'group' is an analysis of identical markers).
- [x] All plots are contour plots with outliers or pseudocolor plots.
- [x] A numerical value for number of cells or percentage (with statistics) is provided.

Methodology

Sample preparation	Cells were prepared for flow cytometry by trypsinization followed by re-suspension in FACS buffer.
Instrument	Samples were collected on a BD LSR Fortessa.
Software	FACSDiva version 8.0.1 software was used to collect flow cytometry data. Custom MATLAB code was used for analysis.
Cell population abundance	Sorted cells were single-cell sorted and therefore do not represent an abundance of the population. All construct types were sorted using the same copy number gate (EBFP) with the same mode. While, post-sort EBFP fluorescence was measured at each time point, we expected drifts in EBFP fluorescence due to epigenetic silencing thus this is not a purity metric.
Gating strategy	Morphological gating was performed by gating on forward scatter (FSC) vs side scatter (SSC) area measurements to separate cells from debris, while additional gates on FSC and SSC height vs width measurements isolated singlet cells. Where it was necessary to gate untransfected cells from positively-transfected cells or highly-transfected cells, gate values are provided and example plots depicting gates are shown.

- [x] Tick this box to confirm that a figure exemplifying the gating strategy is provided in the Supplementary Information.
