# Supplementary material for: PERSIST platform provides programmable RNA regulation using CRISPR endoRNases
Source: Nat Commun. 2022 May 11;13:2582. doi: 10.1038/s41467-022-30172-3 (PMC9095627; doi:10.1038/s41467-022-30172-3)
Supplement: Supplementary file 4 — Description of Additional Supplementary Files [file 41467_2022_30172_MOESM4_ESM.pdf]

Title: Supplementary Data 1

Description: This file includes sequences of plasmid parts and a list of all plasmids used in the study with associated descriptions.
